# Behavioural and physiological responses of laying hens to automated monitoring equipment

**DOI:** 10.1016/j.applanim.2017.10.017

**Published:** 2018-02

**Authors:** Stephanie Buijs, Francesca Booth, Gemma Richards, Laura McGaughey, Christine J. Nicol, Joanne Edgar, John F. Tarlton

**Affiliations:** a School of Veterinary Sciences, University of Bristol, Langford house, Langford, BS40 5DU, United Kingdom; b Royal Veterinary College, Hawkshead Lane, Hatfield, AL9 7TA, United Kingdom

**Keywords:** Automated monitoring, Aggression, Preening, Thermography, Domestic fowl

## Abstract

Automated monitoring of behaviour can offer a wealth of information in circumstances where observing behaviour is difficult or time consuming. However, this often requires attaching monitoring devices to the animal which can alter behaviour, potentially invalidating any data collected. Birds often show increased preening and energy expenditure when wearing devices and, especially in laying hens, there is a risk that individuals wearing devices will attract aggression from conspecifics. We studied the behavioural and physiological response of 20 laying hens to backpacks containing monitoring devices fastened with elastic loops around the wing base. We hypothesised that backpacks would lead to a stress-induced decrease in peripheral temperature, increased preening, more aggression from conspecifics, and reduced bodyweights. This was evaluated by thermography of the eye and comb (when isolated after fitting backpacks), direct observations of behaviour (when isolated, when placed back into the group, and on later days), and weighing (before and after each 7-day experimental period). Each hen wore a backpack during one of the two experimental periods only and was used as her own control. Contrary to our hypothesis, eye temperature was higher when hens wore a backpack (No backpack: 30.2 °C (IQR: 29.0–30.6) vs. Backpack: 30.9 °C (IQR: 30.0–32.0), P < 0.001). Eye temperature of hens wearing a backpack was strongly correlated to the time spent preening (r_s_ = 0.8, P < 0.001), suggesting that the higher temperatures may have been due to preening itself, or to a low head position or decreased heat dissipation when preening under the wings. Aggressive behaviour was very rare and no effect of the backpacks was found. In line with our hypothesis, backpacks increased preening on the day of fitting, both when isolated (No backpack: 0% (IQR: 0–1) vs. Backpack: 22% (IQR: 1–43), P < 0.01) and when back in the group (No backpack: 0% (IQR: 0–27) vs. Backpack: 43% (IQR: 5–77), P < 0.001). However, no effect on preening was observed 2–7 days afterwards. Other behavioural changes suggested that on the day of fitting hens prioritized attempts to (re)move the backpack and were less attentive to their surroundings. However, only equipment pecking (i.e., pecking the backpack or leg rings) was still affected 2–7 days after fitting (No backpack: 0 pecks/hen/minute (IQR: 0–0), vs. Backpack: 0 (IQR: 0–0.07), P < 0.05). We found no effect of our backpacks on bodyweight. In conclusion, our backpacks seem suitable to attach monitoring equipment to hens with only a very minor effect on their behaviour after a short acclimation period (≤2 days).

## Highlights

• Monitoring devices affected adult hen behaviour on the day of fitting.
• Hens prioritized (re)moving newly fitted devices over exploration.
• Devices did not increase aggressive behaviour towards equipped hens.
• From two days after fitting on, only a very minor effect on behaviour was observed.
• Peripheral eye temperature seemed related to preening behaviour rather than stress.

## 1 Introduction

Automated technology is increasingly used to monitor animal behaviour (Barron et al., 2010, Siegford et al., 2016). It allows efficient continuous data collection from many individuals simultaneously, in situations where human observations are inconvenient (e.g., at night), difficult (e.g. when focal animals are hard to discern or reach), or may disturb behaviour. In addition, automation may eliminate certain types of observation bias (Marsh and Hanlon, 2004). However, except for technologies that do not differentiate between individuals or are purely video-based, automated monitoring necessitates the attachment of monitoring devices to animals. This can alter behaviour, and even invalidate the data collected. Monitoring devices increase energy expenditure, decrease foraging and increase preening in several free-living bird species (Barron et al., 2010). Such effects occur in species that primarily walk as well as in species that primarily fly and are therefore likely to apply to laying hens. Hens wearing monitoring devices may also attract aggression from their conspecifics, as devices usually alter their appearance. Even minor changes in appearance can attract aggression and lead to decreased bodyweights and altered adrenaline and dopamine levels (Dennis et al., 2008, Liste et al., 2015, Campderrich et al., 2017). Chickens also peck each other during social exploration (Riedstra and Groothuis, 2002) and equipment may renew the motivation for such exploration, increasing the number of pecks received.

No previous studies have assessed whether adult chickens adapt their behaviour when fitted with devices for automated behavioural monitoring, and only two have assessed this in sub-adults. Daigle et al. (2012) found that devices mounted on the backs of pre-lay pullets led to short-term decreased feeder and drinker use, whilst increasing perch and nest box use. No indications of increased energy expenditure or agonistic behaviour were found 17 and 8 days after device fitting, respectively. In slow-growing broiler chickens wearing back-mounted devices, walking and pecking was affected in the week after fitting only (Stadig et al., 2017). Although this suggests that there are no long-term effects, short-term effects are also of interest, as often the intention is to collect data shortly after fitting. Crucially, literature on wild birds (Barron et al., 2010) suggests that behaviours not included in previous studies, such as time spent preening, may be affected. Also, effects on adult laying hens may substantially differ from those observed in young chickens.

In our pilot studies we used ‘vests’ of stretchy fabric or plastic cases in contrasting colours to fit devices and observed immediate marked responses including sidestepping/reversing (interpreted as attempts to escape from underneath the equipment), running away in apparent panic, and simply lying down during the first 15 min after fitting. Several days later hens still pecked or pulled the devices frequently, were often attacked and chased by conspecifics, and were seen to isolate themselves in nest boxes or on perches. We therefore developed a less visible and obtrusive attachment system. This consisted of a ‘backpack’ only slightly larger than the devices contained, with smooth angles and in the same colour as the hen, which was attached by elastic loops around the base of the wings. In a small scale trial on a commercial farm (Buijs et al., 2017), these backpacks had only a minor effect on behaviour (i.e., equipped hens received slightly more pecks but did not show other significant differences in behaviour). The current study was designed to systematically evaluate the behavioural and physiological response to these backpacks.

We hypothesised that if our backpacks would not be well tolerated, hens would increase the time they spent preening, sidestepping/reversing, sitting/lying, and the frequency of equipment pecking (i.e., pecking the backpack and leg rings, the latter being fitted continuously on all hens for identification purposes). We also hypothesised that hens wearing a backpack would be pecked and attacked more often, leading to increased plumage damage and attempts to withdraw by fleeing, perching, or hiding in the nest box. This was predicted to reduce foraging and eating/drinking resulting in lower body weights.

Physiological responses shortly after fitting the backpacks were analysed by infrared thermography, a non-invasive indicator of arousal. Acute stress leads to an initial decrease in peripheral temperature due to vasoconstriction (Cabanac and Aizawa, 2000, Moe et al., 2012). Mild stressors like handling and air puffs reduce comb, wattle and eye temperature (Edgar et al., 2011, Edgar et al., 2013, Herborn et al., 2015), although reward-downshift or more difficult decisions do not (Davies et al., 2014, Davies et al., 2015). We hypothesised that backpacks would reduce peripheral temperature. Peripheral temperature can also drop in situations that are likely to be positively valanced (Moe et al., 2012) but, in combination with behaviour supposedly aimed at removing the backpack, we would interpret fitting as an aversive experience. Defaecation rate was used as a second stress indicator (Hall, 1934, de Haas et al., 2010) and was hypothesised to be higher when wearing a backpack.

## 2 Methods

The study was carried out following ethical approval by the University of Bristol (license number UB/17/002).

### 2.1 Animals and housing

Twenty 18-week-old British Blacktail laying hens were obtained from a commercial rearing farm and transported to the test facility after weighing and fitting leg rings for individual identification. All hens were housed together throughout, in a 13.8 m^2^ floor pen covered with wood shavings. Hens had continuous access to commercial layer mash, water, a three-tier perch, nest boxes, a slatted ramp and environmental enrichment (an alfalfa bale and pecking block), except when put in the holding pen (2 × 5 min per hen in total). Room temperature was maintained between 16 and 19 °C throughout the study.

In the week before data collection the hens were habituated to human presence. In addition to the normal exposure to humans during routine husbandry procedures (replacing feed and water and egg collection), at least one person was present in the house during most of the light period. During the first two days of habituation she moved around the pen freely, but did not enter the pen. On the third and fourth day she entered the pen, but did not actively approach any of the hens. Hens that approached her calmly were picked up briefly and placed back on the floor immediately and carefully. All hens had allowed this by the end of the fourth day and none showed clear avoidance behaviour after being picked up. Two days before the experiment started all hens were picked up, handled, weighed, mite-treated, and put back in the pen.

### 2.2 Fitting backpacks

**Fig. 1:**
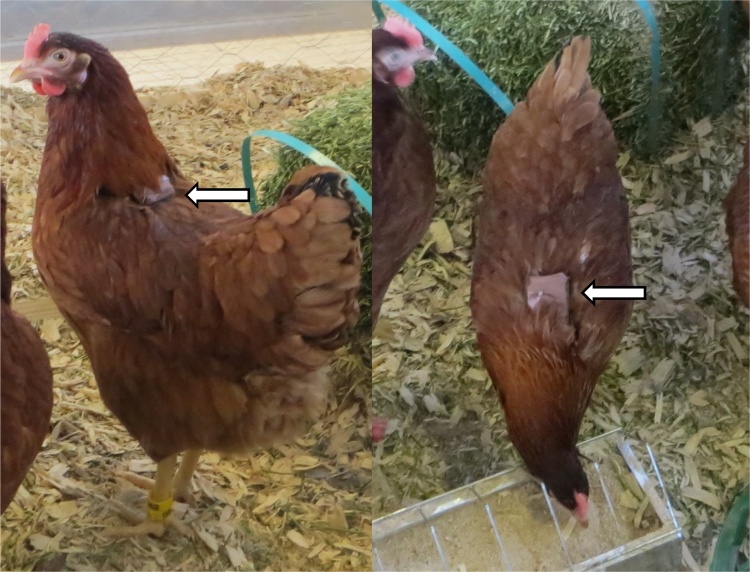
Arrows indicate the backpack containing the equipment as visible when standing up and bending down.

Each hen was fitted with an approximately 50 g backpack when 23–24 weeks old. Each backpack contained three monitoring devices intended for later studies: a light sensor (Biotrack Ltd, Wareham, United Kingdom), tri-axial accelerometer (Custom Idea Ltd, Shepton Mallet, UK), and a location device (Tile Mate, Tile Inc., San Mateo, United States). The equipment was wrapped in brown electrical tape and attached to the back of the hen using elastic loops around the wing base. This meant that the larger part of the package was covered by the neck feathers when the head was up (Fig. 1). On day 0 (five weeks after arrival at the test facility) half of the hens received backpacks, which were removed at the end of day 7. On day 8 the other half of the hens received backpacks, which they wore until day 15. These two groups were balanced for initial body weight and the order in which they had been caught at the rearing farm (a possible indicator of fear of humans).

Hens that belonged to the group that was not fitted with backpacks on a certain day were instead held by an experimenter for 30 s (the approximate amount of time it took to put on a backpack) before the start of data collection (i.e., the first thermal image). To avoid confounding between our treatment and possible circadian patterns, hens that were to be equipped on that day and those that were not were collected from the pen alternatingly. As hens would be used as their own control (comparing their behaviour and peripheral temperature when equipped to values when not equipped) individuals were handled in the same order on day 8 as on day 0.

### 2.3 Thermography

Immediately after being equipped or held, each hen was held and photographed (in profile orientation) at a standardized location 1 m from the FLIR E50bx thermal camera (emissivity: 0.96). One minute later a second thermal image was taken after which the hen was placed in a holding pen (91 × 62 × 62 cm length × width × height) which permitted visual, but not physical, contact with conspecifics. Whilst in the pen five thermal images were taken at approximate 1 min intervals from a standardized distance, attempting to photograph the hen in profile and when not moving. Hens were photographed away from metal surfaces and air currents (which distort thermal images). Because acquiring reliable images (of immobile hens away from metal and air currents and at a standardized distance) was not possible in the home pen, no thermal images were taken before collecting the hen, after placing the hen back, or on subsequent days.

Thermal images were analysed using the Thermacam Reporter Professional 2000 software package (FLIR, Wilsonville, Oregon), acquiring the temperature of the midpoint of the eye and an average temperature within the traced area of the comb.

### 2.4 Behaviour

Behaviour was assessed using direct continuous focal observation. Each hen was observed during the five minutes she spent in the holding pen and during the first five minutes after release from the holding pen back into the home pen. In addition, all hens were observed 2, 3, 5, 7 days after each day of backpack fitting for 10 min per hen per day. Hens were always observed in the same order in which they had been handled during backpack fitting.

All observations were made by a single observer using Obansys software (Mangold International, Arnstorf, Germany) on a handheld device. The percentage of observed time spent sitting/lying, standing, walking, sidestepping/reversing, foraging, preening, eating/drinking, dustbathing and performing and receiving gentle feather pecks was recorded, simultaneously scoring the percentage of time spent in different locations (floor, ramp, perch and nest box). In addition, the frequency of agonistic behaviour (head pecks and claws given or received, stand-offs, fights, fleeing), body pecks, severe feather pecks (both performed and received for all types of pecks), wing flaps and wall pecks was simultaneously recorded, as was the frequency of equipment pecks (to the backpack or leg rings if hens were wearing a backpack and to the leg rings only when hens were not wearing a backpack). We did not distinguish between ring pecks and backpack pecks, as we would not consider a shift from pecking one type of equipment to another as a reason not to recommend the use of backpacks in further research.

### 2.5 Faeces

Any faeces produced whilst the hen was in the holding pen or whilst being weighed seven days later were noted. Faeces were collected and stored with the intention to analyse glucocorticoid metabolites.

### 2.6 Bodyweight and plumage score

Individual bodyweights and plumage damage were assessed one day before and 7 days after fitting the backpacks (and handling the hens that did not receive a backpack in that week). These were recalculated into weight gain (or loss) and increase in plumage damage.

Plumage damage was assessed separately in three areas (head/neck, tail tip, vent/underside) on a 3-point scale (0: no bare skin visible, no or very light damage, only one feather broken/missing, 1: mild wear on feathers, several damaged/frayed feathers together or two or more broken/missing feathers, bare skin visible <5 cm^2^, 2: bare skin visible ≥5 cm^2^). Plumage damage to the back was not scored as the backpack covered part of this area.

### 2.7 Statistical analysis

As data often showed a non-normal distribution we used non-parametric analyses throughout. For the analyses of behaviour, peripheral temperature and bodyweight gain hens were used as their own controls, comparing results when wearing the backpack to results when not wearing it using Wilcoxon signed rank tests. Behaviour was analysed separately for the data collected in the holding pen and the home pen on the day of fitting, and for each of the days after fitting. In addition, the data collected on days 2–7 after fitting were averaged and analysed as described above. Temperature was analysed separately at each time point. In addition, the average value of the five temperatures whilst in the holding pen was analysed. Defaecation rates were analysed using Fisher’s exact test.

Batch effects (e.g., differences between hens wearing backpack in the first (day 0–7) and in the second (day 8–15) experimental period) were analysed using Wilcoxon rank sum tests. Correlations between behaviour and eye temperature in the holding pen were analysed using Spearman rank correlation tests. These analyses were done separately for hens with and without a backpack.

## 3 Results

### 3.1 Behaviour

Throughout the experiment, agonistic behaviour other than head pecking was extremely rare. Focal hens were never observed to flee or claw, engaged in only one fight and two stand-offs, and were clawed by a conspecific only twice. Similarly, performing and receiving severe feather pecking was very rare (3 and 2 occurrences, respectively, from a total of 30 h observation). Because of their low occurrence none of these behaviours were analysed statistically.

Newly backpacked hens placed in the holding pen spent significantly more time preening, sitting/lying and sidestepping/reversing, but less time standing and walking than when they were placed into the holding pen without a backpack (Table 1). When newly backpacked, hens also pecked their equipment (i.e., their backpack or leg rings) more, whilst pecking the pen less, than when placed in the holding pen without a backpack. After the hens were moved from the holding pen to the home pen on the day of fitting, the effect of the backpack on sitting/lying, standing and wall pecking no longer reached significance, whilst the effect on sidestepping/reversing was reduced to a tendency. However, hens still preened and pecked their equipment (i.e., backpack or leg rings) more when wearing the backpack than when without and walked and foraged less. In addition, hens showed differences in behaviours not possible in the holding pen: when wearing the backpack hens ate/drank less and received more equipment pecks from other hens than when without.

**Table 1:** Behaviour of laying hens when either equipped with a backpack or not (medians + interquartile ranges). Significant differences and tendencies as shown by Wilcoxon signed rank tests indicated in bold. *** P < 0.001, ** P < 0.01, * P < 0.05, ^#^ P < 0.10. – Behaviour not possible in this situation. Medians displayed without a Z-score indicate that the behaviour was possible, but now shown. GFP: Gentle feather peck.

	Day of equipping – Holding pen			Day of equipping − Home pen			2–7 days after equipping		
	No pack	Backpack	Z	No pack	Backpack	Z	No pack	Backpack	Z
% of observed time									
Stand	**70 (57–76)**	28 (15–52)***	**3.5**	9 (5–14)	11 (7–37)	−1.3	15 (12–18)	14 (9–23)	0.4
Walk	**28 (23–35)**	15 (5–23)*	**2.5**	**21 (14–35)**	8 (2–13)**	**2.9**	8 (5–12)	8 (4–12)	1.5
Preen	**0 (0–1)**	22 (1–43)**	**−2.9**	**0 (0–27)**	43 (5–77)***	**−3.8**	9 (5–21)	13 (3–24)	0.4
Sit or lie	**0 (0–0)**	0 (0−32)**	**−2.7**	0 (0–0)	0 (0–0)	−0.4	4 (2–8)	3 (1–6)	1.1
Sidestep or reverse	**0 (0–0)**	1 (0–9)***	**−3.4**	**0 (0–0)**	**0 (0–0)^#^**	**−1.8**	0 (0–0)	0 (0–0)	−1.0
Forage	0 (0–0)	0 (0–0)	.	**8 (0–29)**	0 (0–0)*	**2.2**	31 (19–42)	38 (25–44)	−1.3
Dustbathe	0 (0–0)	0 (0–0)	.	0 (0–0)	0 (0–0)	−1.6	0 (0–4)	0 (0–3)	−0.4
Eat or drink	–	–	.	**6 (1–66)**	0 (0–10)*	**2.1**	16 (9–22)	12 (9–22)	0.4
Gentle feather peck	–	–	.	0 (0–0)	0 (0–0)	.	0 (0–0)	0 (0–0)	−1.6
Receive GFP^1^	–	–	.	0 (0–0)	0 (0–0)	.	0 (0–0)	0 (0–0)	−0.7
Floor	–	–	.	100 (100–100)	100 (100–100)	−1.3	99 (96–100)	99 (95–100)	−0.7
Perch	–	–	.	0 (0–0)	0 (0–0)	.	0 (0–0)	0 (0–0)	1.4
Ramp	–	–	.	0 (0–0)	0 (0–0)	.	0 (0–1)	0 (0–1)	−0.5
Nest box	–	–	.	0 (0–0)	0 (0–0)	1.3	0 (0–0)	0 (0–0)	−1.0

Frequency (#/hen/min)									
Wall peck	**3 (1–4)**	1 (0–2)**	**2.6**	0 (0–0.1)	0 (0–0)	1.3	0.01 (0–0.15)	0.02 (0–0.11)	0.1
Peck equipment	**0 (0–0)**	3 (0–6)***	**−3.4**	**0 (0–0)**	7 (4–10)***	**−3.8**	**0 (0–0)**	0 (0–0.07)*	**−2.5**
Jump or fly	0 (0–0)	0 (0–0)	.	0 (0–0)	0 (0–0)	0.7	0.05 (0.02–0.07)	0.02 (0–0.05)	1.6
Body peck	0 (0–0)	0 (0–0)	.	0 (0–0)	0 (0–0)	−1.0	0 (0–0.01)	0 (0–0.01)	−1.1
Wing flap	0 (0–0)	0 (0–0)	−0.4	0 (0–0)	0 (0–0)	.	0 (0–0)	0 (0–0)	0.0
Stretch	0 (0–0)	0 (0–0)	.	0 (0–0)	0 (0–0)	1.3	0.02 (0–0.02)	0.01 (0–0.05)	−0.7
Head peck	–	–	.	0 (0–0)	0 (0–0)	−1.3	0.01 (0–0.10)	0 (0–0.06)	−1.5
Receive equipment peck	–	–	.	**0 (0–0)**	0 (0−2)*	**−2.3**	0 (0–0)	0 (0–0)	−1.3
Receive head peck	–	–	.	0 (0–0)	0 (0–0)	0.4	0 (0–0.05)	0 (0–0.08)	−1.2
Receive body peck	–	–	.	0 (0–0)	0 (0–0)	−1.3	0 (0–0.01)	0 (0–0.02)	−0.7

Two days after fitting the backpacks, hens jumped or flew less and pecked their equipment more when they wore a backpack, although these effects were minor and displayed by a minority of the hens (jump/fly – Backpack: 0 (IQR: 0–0) vs. No backpack: 0 (IQR: 0-0.1), P < 0.05, Z = 2.2, peck equipment – Backpack: 0 (IQR: 0–0.12) vs. No backpack: 0 (IQR: 0–0), P < 0.05, Z = −2.4). Three days after fitting, hens received more feather pecks when they wore a backpack (Backpack: 0 (IQR: 0–0) vs. No backpack: 0 (IQR: 0–0), P < 0.05, Z = −2.0). Seven days after fitting hens pecked their equipment more when wearing a backpack (Backpack: 0 (IQR: 0–0.1) vs. No backpack: 0 (IQR: 0–0), P < 0.05, Z = −2.0). No other significant effects were detected on days 2, 3, 5 and 7 (Supplementary Tables 1 and 2). Because the effect of the backpacks thus seemed far subtler on the days after (rather than immediately after) fitting, data from these four days were averaged to acquire a more robust estimate of behaviour per hen. Analysis of this averaged data showed that when wearing a backpack, hens pecked their equipment significantly more often (Table 1).

Behaviour whilst wearing a backpack was also influenced by whether the backpack was worn in the first (day 0–7) or the second (day 8-15) experimental period (Table 2). Compared to hens wearing a backpack in the first period of the experiment, hens wearing a backpack in the second period preened more whilst in the holding pen, and received fewer pecks and stood and walked less after release into the home pen. Hens that wore a backpack in the second period also spent less time on gentle feather pecking and standing, spent less time on the ramp, received fewer equipment pecks and performed fewer body pecks 2–7 days after equipping than hens that received a backpack in the first period. Behaviour whilst not wearing a backpack also showed some batch effects, mainly on the day of fitting. Hens that went without a backpack in the second period of the experiment pecked the walls less often (both in the holding pen and the home pen) and walked less and ate more after release into the home pen than hens that went without a backpack in the first batch. Hens that went without a backpack in the second period of the experiment walked more during day 2–7 than those that went without a backpack in the first period.

**Table 2:** Effects of the experimental half on behaviour and physiology (medians + interquartile ranges). Only measures for which a significant effect was found using a Wilcoxon rank sum tests are shown, another 122 measure × situation × treatment combinations were tested but no significant batch effect was found. *** P < 0.001, ** P < 0.01, * P < 0.05.

Back pack	Situation	Measure	First period (day 0–7)	Second period (day 8–15)	Z
Yes	Holding pen	% time spent preening	3 (0–24)	45 (17–56)*	−2.5
	Home pen – Immediately after release	Equipment pecks received/min	2.0 (0–3.1)	0 (0–0)*	2.4
		% time standing	39 (24–49)	7 (7–9)***	3.8
		% time walking	11 (7–16)	3 (2–9)*	2.0
	Home pen – 2–7 days after equipping	Body pecks received/min	0.02 (0–0.05)	0 (0–0)*	2.5
		Equipment pecks received/min	0 (0–0.02)	0 (0–0)*	2.2
		% time feather pecking (gentle)	0 (0–0.12)	0 (0–0)*	2.2
		% time standing	19 (10–21)	9 (6–12)*	2.0
		% time on ramp	0.2 (0–1.5)	0 (0–0)*	2.0
	0–7 days after equipping	Weight gain (kg)	0.19 (0.16–0.23)	−0.01 (−0.03–0.06)**	2.7
No	Holding pen	Wall pecks/min	3.9 (3.2–4.7)	1.1 (0.1–2.3)***	3.4
	Home pen – Immediately after release	Wall pecks/min	0.2 (0–0.4)	0 (0–0)*	2.4
		% time walking	35 (22–56)	17 (12–21)*	2.3
		% time eating/drinking	3 (0–8)	66 (8–75)*	−2.1
	Home pen – 2–7 days after equipping	% time walking	7 (5–10)	11 (8–13)*	−2.0
	0–7 days after equipping	Weight gain (kg)	0.19 (0.17–0.22)	0.04 (0.01–0.07)**	3.0
	Immediately after equipping	Eye temperature	30.1 (29.9–30.7)	31.0 (30.3–31.6)*	−2.3

The hens had usually all laid their egg before the observations started, and no clear signs of pre-lay behaviour were observed. In line with this, nestbox use was rare (Table 1).

### 3.2 Peripheral temperature

Tracing the outline of the comb on the thermal images taken when the hens were in the holding pen proved difficult, as the comb was often partly obscured by the bars of the pen. Therefore, the comb images when in the holding pen were discarded. In addition, 11 out of 40 comb images and 29 out of 120 eye images had to be discarded due to poor image quality.

Comb and eye temperature taken directly and one minute after handling did not differ when hens had been equipped vs. when they were held only (P = 0.32–0.84, Z = −1.0–0.2). However, average eye temperature whilst in the holding pen was significantly higher when hens were wearing a backpack (No backpack: 30.2 °C (29.0–30.6) vs. Backpack: 30.9 °C (30.0–32.0), P < 0.001, Z = −3.7).

**Fig. 2:**
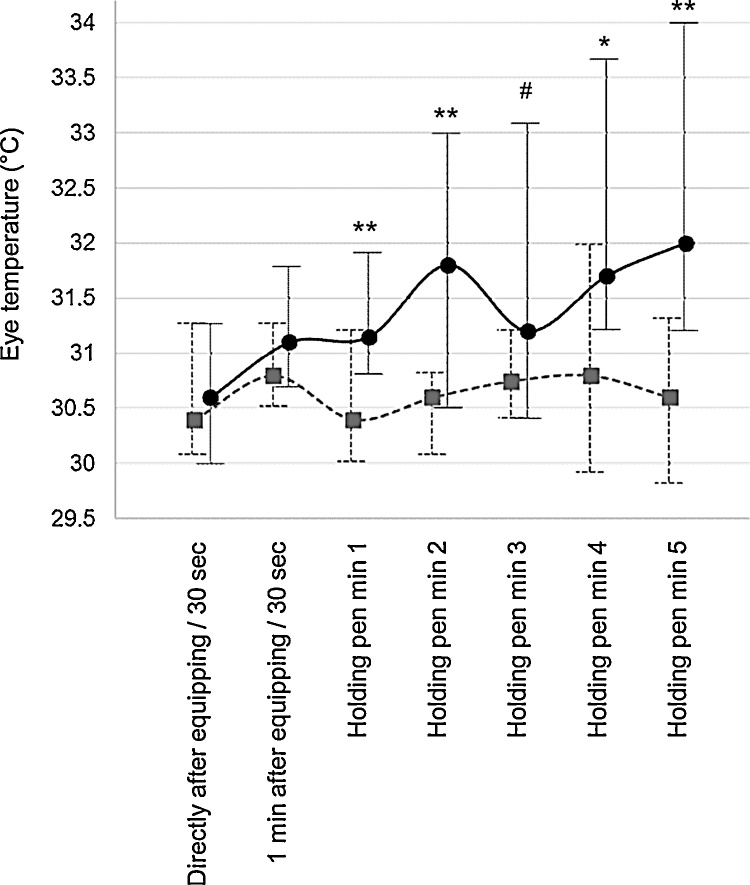
Medians + interquartile ranges of eye temperature of hens after equipping with a backpack (solid black ●) or being held (dotted grey ). Significant differences and trends as shown by Wilcoxon signed rank tests: *** P < 0.001, ** P < 0.01, * P < 0.05, ^#^ P < 0.10. Although results from backpacked and non-backpacked hens were obtained within the same sessions, results for the hens that were equipped with backpacks are shown slightly to the right for visualization purposes.

More detailed analysis of the pattern of eye temperature over time showed that eye temperature was significantly higher (or tended to be) when the hen was wearing a backpack than when she was not, for all minutes in the holding pen (Fig. 2, Z-scores for minute 1–5: −2.8, −2.6, −1.9, −2.2, −3.1).

The hens that did not receive a backpack during the first period (day 0–7) of the experiment had a lower eye temperature 30 s after retrieval from the pen than those that did not receive a backpack in the second period (day 8–15) of the experiment (Table 2). No batch effect was found for hens whilst wearing a backpack (P > 0.05).

### 3.3 Correlation between behaviour and eye temperature

When hens were wearing a backpack, their average eye temperature whilst in the holding pen was strongly correlated to the percentage of time they spent preening (r_s_ = 0.78, P < 0.001). A similar association was found when not wearing the backpack, although in this case it was less expressed (r_s_ = 0.48, P = 0.03).

In addition, eye temperature of hens wearing a backpack was positively associated with the number of equipment pecks (r_s_ = 0.52, P = 0.019) and negatively associated with time spent sidestepping/reversing (r_s_ = −0.61, P = 0.004) and sitting/lying (r_s_ = −0.56, P = 0.011). These behaviours were rare to absent in hens not wearing a backpack though, and no association was found in this subset.

### 3.4 Faeces and plumage damage

Less than a third of the hens defaecated in the holding pen whilst wearing a backpack and none of the hens defaecated whilst being weighed. Therefore, no glucocorticoid analysis was performed. Defaecation rate was significantly lower when hens wore backpacks than when without (6 vs. 18, P < 0.0001). The two hens that did not defecate when not wearing a backpack did not do so when wearing the backpack either. Defaecation rate was 3 out of 10 for backpacked hens and 9 out of 10 for non-backpacked hens in both halves of the experiment.

Only two hens showed any plumage damage (one when wearing a backpack and one when without) and therefore plumage damage was not analysed statistically.

### 3.5 Bodyweight

Bodyweight increased during the experiment. More specifically, hens gained weight in the first period of the experiment rather than in the second period, regardless of the half of the experiment during which they wore a backpack (Table 2).

Median body weight gain during the 7-day period when wearing the backpack was not found to differ significantly from weight gain during the 7-day period when not wearing the backpack (No backpack: 110 g (IQR: 28–190) vs. Backpack: 70 g (IQR: 10–195), P = 0.82, Z = 0.2).

## 4 Discussion

We aimed to assess how laying hens responded (behaviourally and physiologically) to being fitted with a backpack containing measuring devices, as studies using such hen-mounted devices are gaining popularity.

Agonistic behaviour was very rare throughout the study, which was an important finding as in our pilot studies (on several commercial flocks of the same breed though slightly older than the ones used in the present study) hens with more discernible devices had frequently been attacked and chased, which distorted their behaviour. In the current study the only elements of agonistic behaviour that occurred frequently enough for analysis (receiving and performing head pecks) did not increase significantly when hens wore backpacks, in line with previous observations in pullets (Daigle et al., 2012). Feather pecking was also very rare in our study, and together with the low occurrence of agonistic behaviour this explains why almost no plumage damage occurred. Hens with and without backpacks were housed together. Thus, changes in behaviour involving the hens with backpacks could theoretically have resulted in similar changes in the hens without backpacks (thus, obscuring treatment differences). However, because agonistic behaviour and feather pecking were so rare in this study this seems unlikely. Laying hen behaviour (especially agonistic behaviour) can be affected by group size (D’Eath and Keeling, 2003), which limits the extent to which our results can be extrapolated directly to larger groups (for instance to commercial flocks). However, in our previous small scale trial on a commercial 2000 hen flock (Buijs et al., 2017) we observed a similar lack of impact on behaviour, suggesting that the results obtained can be extrapolated.

Although agonistic behaviour was very rare, the hens did show other changes in behaviour when wearing our backpacks. In line with our expectations, newly equipped hens spent more time performing behaviours that likely reflect attempts to remove the backpack or to move it to a more comfortable position (i.e., sidestepping/reversing, preening, equipment pecking) when placed in the holding pen. Conversely, hens seemed less interested in exploring or escaping the holding pen when wearing a backpack, as they spent less time walking and pecked the pen less. Most of these differences were also observed in hens that had been placed back in the home pen, where hens also ate/drank and foraged less and received more equipment pecks. These equipment pecks were mainly received by hens wearing the backpacks during the first period of the experiment (day 0–7), suggesting that hens became less interested in the backpacks upon repeated exposure. A quarter of the hens spent most of their time sitting or lying down in the holding pen when wearing a backpack, whilst no hens sat or lay down in the holding pen when not wearing a backpack. In the context of the open-field test, more time spent sitting still is interpreted as a sign of increased fear (Jones, 1992). Our holding pen was not intended as an open-field setup, and in contrast to this test, allowed visual and auditory contact with conspecifics. However, our procedures were similar to the open-field test in that the hen was removed from the flock and placed into an unknown enclosure individually. The high amount of time some of the hens spent sitting and lying when wearing a backpack may therefore mean that these hens were more frightened. However, the markedly lower defaecation rate when wearing a backpack would argue against this, as a low defaecation rate is indicative of decreased fear during the open-field test (Hall, 1934, de Haas et al., 2010). Although behaviour on the day the backpacks were fitted suggested that the hens gave considerable priority to attempting to remove or relocate the backpack, the backpacks had only a very minor effect on their behaviour during later days. Only a slightly increased equipment pecking rate when wearing the backpack was found throughout day 2–7. It seems unsurprising that hens that received more equipment (i.e., a backpack as well as leg rings, instead of leg rings only) would interact with their equipment more often. What is important here is that this behaviour was not displayed in an excessive manner by hens wearing backpacks (in fact, less than half of the hens wearing a backpack was observed to perform such behaviour at all 2–7 days after fitting) and that it did not impact on the time allocated to other behaviours. In addition, bodyweights showed no evidence of the increased energy expenditure previously observed in free-living birds (Barron et al., 2010).

If the backpack or the fitting procedure induced fear, a lower eye temperature would be expected, as hens are reported to show an immediate decrease in peripheral temperature as an initial response to stress (Edgar et al., 2011, Edgar et al., 2013). However, eye temperature after fitting was not lower when hens were equipped than when they were only held. This is in line with Herborn et al. (2015), who found no proportional relation between stressor intensity and eye temperature (although they did observe proportional relations between stressor intensity and wattle and comb temperature). We did observe differences in eye temperature after the hens had been put into the holding pen. In contrast to our expectations, eye temperatures were higher when wearing a backpack. It is unlikely that the lack of a difference before release into the pen was simply due to the time required to mount a thermal response, as previous research (Moe et al., 2012, Edgar et al., 2013) describes effects in the minute after exposure to the stimulus. It seems more likely that the observed differences whilst in the holding pen were due to the behaviour of the hens, which they could only adapt as soon as they were placed into the pen. One of the most prominent responses to the backpack whilst in the holding pen was a high level of preening, which was positively correlated to eye temperature. The straps of the backpack (which looped around the wings) often led to under-wing preening. Keeping the head underneath the wing not only reduces peripheral heat loss (Pickel et al., 2011) but also leads to a relatively low head position. A lower head position was previously found to be associated with higher eye temperatures (Edgar et al., 2013, Herborn et al., 2015). Thus, it seems that the eye temperatures we measured did not reflect a stress response but a direct effect of a change in behaviour. However, preening not only functions to clean and order the feathers but it is also performed after stressful situations, serving as a stress alleviating coping mechanism (Henson et al., 2012). Possibly, when the hens preened to order their feathers after receiving a backpack or being handled, this mitigated the stress induced drop in peripheral temperature as a side effect. Although this is the first study to assess the association between preening and eye temperature directly within treatment, lower eye temperatures have previously been induced by other manipulations that also decreased preening (air puffs: Edgar et al., 2011). This suggests that the association between preening and eye temperature may be broader than a specific response to wearing a backpack and future studies using thermography in poultry would need to take preening behaviour into account.

## 5 Conclusion

The effects of our backpacks on hen behaviour were relatively mild, even nearly absent from 2 days after equipping on. This enables application providing a short period of acclimation is permitted. There was no clear evidence of an overall stress response when studying peripheral temperature and weight gain. This method of attachment thus seems appropriate when fitting technology to monitor laying hen behaviour shortly, though perhaps not immediately, following fitting.
