# Supplementary material for: Behavioural and physiological responses of laying hens to automated monitoring equipment
Source: Appl Anim Behav Sci. 2018 Feb;199:17–23. doi: 10.1016/j.applanim.2017.10.017 (PMC5805850; doi:10.1016/j.applanim.2017.10.017)
Supplement: Supplementary file 1 [file mmc1.docx]

|  |  |  |  | 2 days after equipping | | | | |  | 3 days after equipping | | | |  | 5 days after equipping | | | |  | 7 days after equipping | |
| --- | --- | --- | --- | --- | --- | --- | --- | --- | --- | --- | --- | --- | --- | --- | --- | --- | --- | --- | --- | --- | --- |
|  |  |  |  | No backpack | | Backpack | | |  | No backpack | | Backpack | |  | No backpack | | Backpack | |  | No backpack | Backpack |
| Percentage of observed time | | | | | | |  |  | | |  | |  |  | |  | |  |  |  |  |
|  | Dustbathe |  |  | 0 (0-0) | | 0 (0-0) | | |  | 0 (0-0) | | 0 (0-0)^#^ | |  | 0 (0-0) | | 0 (0-2) | |  | 0 (0-0) | 0 (0-0) |
|  | Sidestep or reverse |  |  | 0 (0-0) | | 0 (0-0) | | |  | 0 (0-0) | | 0 (0-0) | |  | 0 (0-0) | | 0 (0-0) | |  | 0 (0-0) | 0 (0-0) |
|  | Walk |  |  | 4 (0-10) | | 5 (0-12) | | |  | 8 (3-14) | | 4 (2-10) | |  | 6 (3-12) | | 3 (0-10) | |  | 8 (3-13) | 9 (5-12) |
|  | Receive gentle feather peck |  |  | 0 (0-0) | | 0 (0-0) | | |  | **0 (0-0)** | | **0 (0-0)^*^** | |  | 0 (0-0) | | 0 (0-0) | |  | 0 (0-0) | 0 (0-0) |
|  | Gentle feather peck |  |  | 0 (0-0) | | 0 (0-0) | | |  | 0 (0-0) | | 0 (0-0) | |  | 0 (0-0) | | 0 (0-0) | |  | 0 (0-0) | 0 (0-0) |
|  | Eat or drink |  |  | 5 (0-28) | | 6 (0-26) | | |  | 6 (0-31) | | 13 (0-41) | |  | 8 (0-24) | | 4 (0-28) | |  | 12 (3-28) | 3 (0-24) |
|  | Preen |  |  | 1 (0-19) | | 1 (0-33) | | |  | 4 (0-55) | | 3 (0-22) | |  | 0 (0-17) | | 1 (0-5) | |  | 0 (0-3) | 2 (0-20) |
|  | Forage |  |  | 32 (10-42) | | 20 (5-49) | | |  | 17 (3-41) | | 19 (5-52) | |  | 30 (8-54) | | 33 (16-55) | |  | 35 (13-54) | 41 (23-58) |
|  | Sit or lie |  |  | 2 (0-8) | | 0 (0-14) | | |  | 0 (0-2) | | 0 (0-6) | |  | 0 (0-9) | | 0 (0-5) | |  | 0 (0-4) | 0 (0-2) |
|  | Stand |  |  | 14 (6-22) | | 8 (5-11) | | |  | 9 (4-19) | | 10 (4-17) | |  | 12 (5-21) | | 7 (2-19) | |  | 13 (7-23) | 15 (4-27) |
|  | Floor |  |  | 100  (100-100) | | 100  (100-100) | | |  | 100  (100-100) | | 100  (100-100) | |  | 99  (96-100) | | 100  (100-100) | |  | 100  (100-100) | 100  (100-100) |
|  | Perch |  |  | 0 (0-0) | | 0 (0-0) | | |  | 0 (0-0) | | 0 (0-0) | |  | 0 (0-0) | | 0 (0-0) | |  | 0 (0-0) | 0 (0-0) |
|  | Ramp |  |  | 0 (0-0) | | 0 (0-0) | | |  | 0 (0-0) | | 0 (0-0) | |  | 0 (0-0) | | 0 (0-0) | |  | 0 (0-0) | 0 (0-0) |
|  | Nestbox |  |  | 0 (0-0) | | 0 (0-0) | | |  | 0 (0-0) | | 0 (0-0) | |  | 0 (0-0) | | 0 (0-0) | |  | 0 (0-0) | 0 (0-0) |
| Frequency (#/hen/minute) | | | | |  | |  |  | | |  | |  |  | |  | |  |  |  |  |
|  | Jump or fly |  |  | **0 (0-0.1)** | | **0 (0-0)*** | | |  | **0 (0-0.1)** | | **0 (0-0)^#^** | |  | 0 (0-0) | | 0 (0-0) | |  | 0 (0-0) | 0 (0-0.02) |
|  | Receive body peck |  |  | 0 (0-0) | | 0 (0-0) | | |  | 0 (0-0) | | 0 (0-0) | |  | 0 (0-0) | | 0 (0-0) | |  | 0 (0-0) | 0 (0-0) |
|  | Body peck |  |  | 0 (0-0) | | 0 (0-0) | | |  | 0 (0-0) | | 0 (0-0) | |  | 0 (0-0) | | 0 (0-0) | |  | 0 (0-0) | 0 (0-0) |
|  | Wing flap |  |  | 0 (0-0) | | 0 (0-0) | | |  | 0 (0-0) | | 0 (0-0) | |  | 0 (0-0) | | 0 (0-0) | |  | 0 (0-0) | 0 (0-0) |
|  | Receive head peck |  |  | 0 (0-0) | | 0 (0-0) | | |  | 0 (0-0) | | 0 (0-0) | |  | 0 (0-0) | | 0 (0-0) | |  | 0 (0-0) | 0 (0-0) |
|  | Head peck |  |  | 0 (0-0) | | 0 (0-0) | | |  | 0 (0-0) | | 0 (0-0) | |  | 0 (0-0) | | 0 (0-0) | |  | 0 (0-0) | 0 (0-0) |
|  | Stretch |  |  | 0 (0-0.02) | | 0 (0-0.1) | | |  | 0 (0-0) | | 0 (0-0) | |  | 0 (0-0) | | 0 (0-0) | |  | 0 (0-0) | 0 (0-0) |
|  | Wall peck |  |  | 0 (0-0) | | 0 (0-0) | | |  | 0 (0-0) | | 0 (0-0) | |  | 0 (0-0.15) | | 0 (0-0) | |  | 0 (0-0) | 0 (0-0.1) |
|  | Receive equipment peck |  |  | 0 (0-0) | | 0 (0-0) | | |  | 0 (0-0) | | 0 (0-0) | |  | 0 (0-0) | | 0 (0-0) | |  | 0 (0-0) | 0 (0-0) |
|  | Peck equipment |  |  | **0 (0-0)** | | **0 (0-0.12)*** | | |  | 0 (0-0) | | 0 (0-0) | |  | 0 (0-0) | | 0 (0-0) | |  | **0 (0-0)** | **0 (0-0.1)*** |

Supplementary table 1: Behaviour of laying hens when either equipped with a backpack or not (medians + interquartile ranges) on days 2-7 after equipping. Significant differences and tendencies as shown by Wilcoxon signed rank tests indicated in bold. *** P<0.001, ** P<0.01, * P<0.05, ^#^ P<0.10.
