# Supplementary material for: Behavioural and physiological responses of laying hens to automated monitoring equipment
Source: Appl Anim Behav Sci. 2018 Feb;199:17–23. doi: 10.1016/j.applanim.2017.10.017 (PMC5805850; doi:10.1016/j.applanim.2017.10.017)
Supplement: Supplementary file 2 [file mmc2.docx]

|  |  | | | 2 days after |  | 3 days after |  | 5 days after |  | 7 days after |
| --- | --- | --- | --- | --- | --- | --- | --- | --- | --- | --- |
| Percentage of time | | |  |  |  |  |  |  |  |  |
|  | Dustbathe | | | 1.1 |  | -1.8 |  | -1.4 |  | 0 |
|  | Sidestep or reverse | | | . |  | . |  | -1.3 |  | . |
|  | Walk | | | -0.3 |  | 1.2 |  | 1.0 |  | 0.1 |
|  | Receive gentle feather peck | | | . |  | **-2.0** |  | 0.4 |  | 1 |
|  | Gentle feather peck | | | . |  | . |  | 0.4 |  | -0.5 |
|  | Eat or drink | | | -0.1 |  | -0.1 |  | 0.2 |  | 0.6 |
|  | Preen | | | -0.5 |  | 0.9 |  | 0.6 |  | -0.8 |
|  | Forage | | | 0.3 |  | 0.0 |  | -0.1 |  | -1.3 |
|  | Sit or lie | | | -0.5 |  | -1.2 |  | 0.7 |  | 1.1 |
|  | Stand | | | 1.0 |  | 0 |  | 0.8 |  | 0.7 |
|  | Floor | | | 0.1 |  | -0.8 |  | -1.6 |  | 0.1 |
|  | Perch | | | 0.0 |  | 0.4 |  | 1.1 |  | -1.3 |
|  | Ramp | | | -0.4 |  | 0.5 |  | -0.1 |  | 1.1 |
|  | Nestbox | | | -1.0 |  | . |  | 1.6 |  | 1 |
| Frequency | |  | |  |  |  |  |  |  |  |
|  | Jump or fly | | | **2.2** |  | 1.8 |  | 0.3 |  | -0.4 |
|  | Receive body peck | | | -1.3 |  | -0.5 |  | 0.4 |  | 1.1 |
|  | Body peck | | | 0.0 |  | -0.5 |  | -0.4 |  | . |
|  | Wing flap | | | 0.4 |  | 0.4 |  | -1 |  | -1.3 |
|  | Receive head peck | | | -1.2 |  | -1.1 |  | 0.3 |  | 1.6 |
|  | Head peck | | | 0.4 |  | 1.6 |  | 1.2 |  | 1.2 |
|  | Stretch | | | -0.4 |  | 0 |  | 0.1 |  | -0.3 |
|  | Wall peck | | | 1.2 |  | -0.3 |  | 1.5 |  | -1.1 |
|  | Receive equipment peck | | | -1.0 |  | -1.6 |  | . |  | . |
|  | Peck equipment | | | **-2.4** |  | -1.2 |  | -0.5 |  | **-2.0** |

Supplementary table 2: Z-values of the analyses in supplementary table 1.
